# GSA-Tuning IPD Control of a Field-Sensed Magnetic Suspension System

**DOI:** 10.3390/s151229879

**Published:** 2015-12-16

**Authors:** Jen-Hsing Li, Juing-Shian Chiou

**Affiliations:** 1 Department of Electrical Engineering, Kun Shan University, 195 Kunda Road, Yongkang District, Tainan City 710, Taiwan; ljh0906@mail.ksu.edu.tw; 2 Department of Electrical Engineering, Southern Taiwan University of Science and Technology, 1 Nan Ti Street, Yongkang District, Tainan City 710, Taiwan

**Keywords:** current transducer, magnetic field sensor, magnetic suspension system, gravitational search algorithm, IPD control

## Abstract

The purpose of this paper is to propose a GSA-tuning IPD control technique for magnetic suspension systems. An educational demonstration on a magnetic-field sensed magnetic suspension system is examined for effectiveness. For the magnetic-field sensed magnetic suspension system (FSMSS), the current transducer is employed for measuring the electromagnetic coil current, and a Hall effect device is used for detecting the position of the suspended object. To achieve optimal performance, the gravitational search algorithm (GSA) is adopted for tuning the integral-proportional-derivative (IPD) controller. The IPD control includes the specified PD controller and an integrator. The specified PD control is employed for stabilizing the inherently unstable FSMSS, whereas the integral control is utilized for eliminating the steady-state error. The GSA can tune the IPD control parameters to enable optimal FSMSS performance. We achieved excellent results from the simulations and hands-on experiments for the proposed control strategies and structures.

## 1. Introduction

Magnetic suspension systems that use forces of attraction are called suspension techniques and those which use forces of repulsion are called levitation techniques [1]. For simplicity, magnetic suspension systems (MSSs) are also called “magnetic levitation systems” or “electromagnetic suspension systems”. An MSS includes electromagnetic coils, suspended objects, power amplifiers, feedback controllers, and sensors. The feedback controller constantly alters the current sent to electromagnets in order to alter the strength of the magnetic force, after which the stable levitation is maintained. Various sensors have been adopted for measuring MSS signals. For example, current transducers are employed for measuring electromagnetic coil currents. An infrared LED pair is used for detecting a suspended object. Hall Effect elements are utilized for sensing the strength of the magnetic field and also for detecting a suspended object. Magnetic suspension technology is critical for engineering applications. For instance, magnetic levitation (Maglev) trains are most commonly known for their application of magnetic levitation. Moreover, an active magnetic bearing for large turbomachinery entails an engineering application.

MSSs have been adopted for applications in engineering and science education. They are interesting devices for students. The MSS was the control engineering subject of an undergraduate project [2] by Wong in 1986. MSS projects have long been used in control system laboratories. Lundberg *et al.* [3,4] taught analysis and design in undergraduate feedback courses by employing an economical MSS setup. Gibbs and his son [5] created a levitating disco ball that was a miniature MSS for his fifth-grade science fair. In addition to hands-on experiments, they have garnered attention in electromagnetic theory. An MSS is naturally a seed of science.

The present study presents a type of miniature MSS. This MSS is named “field-sensed MSS” (FSMSS) [6] because the magnetic field sensor is adopted for measuring the position of a suspended object. This miniature MSS is used for experimental purposes. The MSS is a nonlinear and unstable system; hence, it is an appropriate device for testing various control techniques. Control techniques that have been adequately developed in this miniature MSS can be extended to related plants. Numerous control methods have been tested for the stability and performance of the MSS, including phase-lead compensation by Wong [2], a mixed linear quadratic regulator/H infinity control by Li and Chiou [6], integral-proportional-derivative (IPD) tracking control by Li [7], and adaptive proportional-integral-derivative (PID) control by Lin *et al.* [8]. 

If the characteristics of the controlled system are unknown, then a PID controller is generally considered the most popular of its type. The PID control was invented on the basis of the development of Sperry’s ship autopilot in 1911 [9]. This control attempts to minimize performance indices by adjusting the controller parameters. The PID controller involves three separate parameters and is accordingly occasionally referred to as the three-term control. The proportional control can reduce the rise time and increase the overshoot. However, the integral control can reduce or eliminate the steady-state error but degrades the stability. By contrast, the derivative control can reduce the overshoot but increases the rise time. However, properly tuning these parameters can yield effective transient and steady-state responses. 

This study proposes the gravitational search algorithm (GSA) for IPD controller tuning. The GSA is a newly developed derivative-free global optimum search algorithm. The application of the IPD control of the MSS is an invention for use in engineering applications. The GSA is categorized as a type of swarm intelligence (SI) [10]. In fact, SI refers to the general set of algorithms. Examples of SI include particle swarm optimization (PSO) [11], ant colony optimization, the bee algorithm, bacterial colony optimization, and the GSA [12]. PSO is widely employed for PID controller optimization [8,13]. The GSA is a recent SI algorithm and was developed by Rashedi *et al.* [12]. This algorithm is based on Newton's laws of gravity and motion. The GSA is heuristic and is not based on any assumptions regarding optimal problems. The GSA iteratively attempts to improve a candidate solution regarding fitness. Therefore, the GSA can iteratively tune the IPD controller to achieve optimal performance (fitness). However, constraints exist in the IPD stability control of the FSMSS. This study investigated this issue in detail.

The remainder of this paper is organized as follows: Section 2 introduces a review of the IPD control of the FSMSS. Section 3 presents the GSA for tuning the IPD controller. Section 4 details the simulations of the proposed scheme. Section 5 provides the experiments and results. Finally, Section 6 offers a conclusion.

## 2. IPD Control of the FSMSS

**Figure 1:**
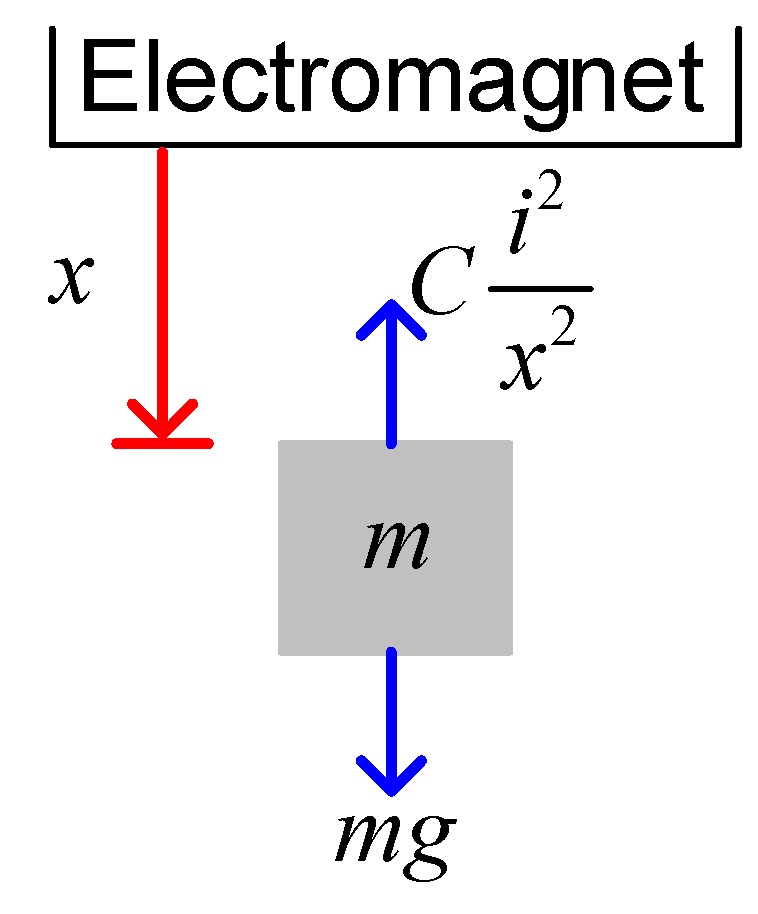
Schematic diagram of an MSS.

Figure 1 shows a one dimensional MSS. The magnet is suspended in the air by the electromagnetic force generated by the electromagnet. Because the influences of other axes (y- and z- axes) are slight enough, it is ignored for dynamics. For simplicity, only vertical axis (x-axis) is considered for discussion. The general analog model of an MSS is given as follows [2,6,7]:

(1) $$m\frac{d^{2}x}{dt^{2}}=mg-C\frac{i^{2}}{x^{2}}$$

where *m* represents the mass of the suspended object, *x* is the distance between the electromagnet and the suspended object, *g* depicts gravitational acceleration, C is the force constant, and i is the electromagnetic coil current. The SI unit of the force constant C is $N\cdot m^{2}/A^{2}$. By electromagnetic theory, the force generated by the electromagnet is $Ci^{2}/x^{2}$ upwards. The gravitational force on the object is *mg* downwards. The friction is neglected in this study. By Newtonian mechanics, Equation (1) is derived and obtained.

The digital model of an MSS is expressed as follows [6,7]:

(2) $$G(z)=\frac{\Delta X(z)}{\Delta I(z)}=\frac{-z\sigma \left(\beta^{2}-1\right)/\beta }{(z-\beta )\left(z-\frac{1}{\beta }\right)}$$

where ΔX(z) is the z transform of Δx, and ΔI(z) represents that of Δi. The parameters are expressed as follows:

(3) $$i_{0}=x_{0}\sqrt{\frac{mg}{C}},\beta =e^{T\sqrt{\frac{2Ci_{0}^{2}}{mx_{o}^{3}}}}>1\mathrm{and}\sigma =\sqrt{\frac{C}{2mx_{o}}}$$

where *T* is the sampling period. Thus, Equation (2) can be modified as follows:

(4) $$\tilde{G}(z)=\frac{\Delta \tilde{X}(z)}{\Delta I(z)}=\frac{\Delta \tilde{X}(z)}{\Delta X(z)}\cdot \frac{\Delta X(z)}{\Delta I(z)}=-\rho \cdot G(z)=\frac{z\sigma \rho \left(\beta^{2}-1\right)/\beta }{(z-\beta )\left(z-\frac{1}{\beta }\right)}=\frac{z\tilde{\sigma }}{z^{2}-\tilde{\beta }z+1}$$

where ρ is the linear factor of the position sensor (Hall Effect device), $\Delta \tilde{X}(z)$ is the z transform of the measured output $\Delta \tilde{x}$ of the position sensor, the parameter $\tilde{\sigma }$ represents $\sigma \rho \left(\beta^{2}-1\right)/\beta$, and $\tilde{\beta }$ depicts $\beta +\beta^{-1}$. The summarized symbols of the FSMSS are listed in Table 1 for a simplified reading of the formulas.

**Figure 2:**
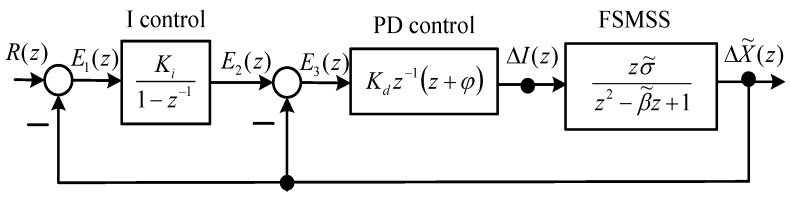
Block diagram of the FSMSS and IPD control.

A block diagram of the tracking IPD control is displayed in Figure 2, and the PD control [6,7] is formulated as follows:

(5) $$G_{pd}(z)=K_{d}z^{-1}(z+\phi )$$

The stable conditions of $K_{d}$ and ϕ were proposed in a previous study [6]. If the initial zero z=−ϕ of the PD control is designed, then the stable range of $K_{d}$ can be obtained using the following formulas:

(6) $$\frac{\left(\beta -1\right)}{\sigma \rho \left(\beta +1\right)\left(1+\phi \right)}<K_{d}<\frac{\left(\beta +1\right)}{\sigma \rho \left(\beta -1\right)\left(1-\phi \right)}$$

and:

(7) $$\frac{-2}{K_{d}\tilde{\sigma }}<\phi <0$$

Although the system parameters are unknown *a priori*, the initially stable settings of *K_d_* and ϕ can be derived easily from a previous study [6]. To eliminate the steady-state error of $\Delta \tilde{x}(k)$, the I control is added. In this case, reference input *R*(*z*) is set to null. If the function of position tracking is enabled, then reference input *R*(*z*) is set properly. The I control can achieve a zero steady-state error. The proper tuning of parameters (*K_d_*, *ϕ*, *K_i_*) can yield effective transient and steady-state responses. The authors of previous studies [6,7] have conducted stability analysis on the IPD control of the MSS. The stability of the overall system is guaranteed when integrator parameter *K_i_* is suitably selected. 

**Table 1:** Symbol summary for a FSMSS.

Symbol	Explanation
m	the mass of the controlled object
g	the gravitational acceleration
C	the force constant
x	the distance between the electromagnet and suspended object
$x_{0}$	the equilibrium position of the suspended object
Δx	$=x-x_{0}$ the deviation of the distance
$\Delta \tilde{x}$	the measured output of the position sensor device
ΔX(s),ΔX(z)	the Laplace transform and z transform of Δx
$\Delta \tilde{X}(z)$	the z transform of the measured output $\Delta \tilde{x}(k)$
i	the coil current
$i_{0}$	the bias current of the equilibrium position
Δi	$=i-i_{0}$ the deviation of the coil current
ΔI(s),ΔI(z)	the Laplace transform and z transform of Δi
T	the sampling period
ρ	the linear factor of the position sensor
β	= $e^{T\sqrt{2Ci_{o}^{2}/mx_{o}^{3}}}$ the derived parameter
$\tilde{\beta }$	= $\beta +\beta^{-1}$ the derived parameter
σ	= $\sqrt{C/2mx_{o}}$ the derived parameter
$\tilde{\sigma }$	= $\sigma \rho \left(\beta^{2}-1\right)/\beta$ the derived parameter
G(z)	the z transform of ΔX(z)/ΔI(z)
$\tilde{G}(z)$	the z transform of $\Delta \tilde{X}(z)/\Delta I(z)$
R(z)	the reference input of Figure 2

## 3. GSA-Tuning IPD Control

The GSA is a derivative-free global optimum search algorithm. It is a type of SI [10] and was originally attributed to Rashedi *et al.* [12]. The GSA is also a nature-inspired algorithm based on Newton's law of gravity and the concept of mass interactions. The searcher agents are a collection of masses. “Agent” is derived from the Latin *agere*. The agent is a computer program that acts as a user or other program in an agency relationship in computer science. Hence, the agent is an autonomous computer program that conducts tasks on behalf of its users.

Each agent has the following four specifications: position, inertial mass, active gravitational mass, and passive gravitational mass. The position of the agent corresponds to a solution of a problem. Gravitational and inertial masses are calculated using a fitting function.

Considering a system with Nm agents, the position of the *i*th agent (agent *i*) can be expressed as follows:

(8) Xi=(xi1,⋯,xid,⋯,xiNd) for i=1,2,⋯,Nm

where $x_{i}^{d}$ is the position of agent *i* in the *d*th dimension, *N_d_* is the dimension of an agent, and *N_m_* is the number of agents. The velocity of agent *i* can be written as follows:

(9) Vi=(vi1,⋯,vid,⋯,viNd) for i=1,2,⋯,Nm

where $v_{i}^{d}$ is the velocity of agent *i* in the *d*th dimension. A gravitational force where agent *j* acts on agent *i* is provided in Equation (10), the concept of which is employed in work that is based on Newtonian gravity and the law of motion. The gravitational force between two particles is directly proportional to the product of their masses and inversely proportional to the square of the distance between them:

(10) $$F_{ij}^{d}(t)=G(t)\frac{M_{pi}(t)\cdot M_{aj}(t)}{R_{ij}(t)+\varepsilon }\left(x_{j}^{d}(t)-x_{i}^{d}(t)\right)$$

where ε is a small positive constant, and ε is introduced to prevent the denominator from being zero. In addition, *M_aj_* represents the active gravitational mass related to agent *j*, and *M_pi_* is the passive gravitational mass related to agent *i*. *G*(*t*) is the gravitational coefficient at time *t* and decreases over time for controlling the search accuracy. *G*(*t*) can be determined as follows:

(11) $$G(t)=G(t_{0})e^{\left(-\alpha \cdot \frac{t}{t_{\max}}\right)}$$

where $G(t_{0})$ is the initial value, α is a positive constant, t is the current iteration, and $t_{\max}$ represents the maximum iteration. $R_{ij}(t)$ is the Euclidian distance between the two agents *i* and *j* and is rewritten as follows:

(12) $$R_{ij}(t)={\left\|X_{i}(t),X_{j}(t)\right\|}_{2}$$

The total force acting on agent *i* in the *d*th dimension is expressed as follows:

(13) $$F_{i}^{d}(t)=\sum_{j\in Kbest,j\neq i}rand_{j}\cdot F_{ij}^{d}(t)$$

where *rand_j_* is a random number in the interval [0,1]. *Kbest* represents the set of first agents with greater mass and the optimal fitness value. Thus, on the basis of the law of motion, the acceleration of agent *i* at time *t* in the *d*th dimension is written as follows:

(14) $$a_{i}^{d}(t)=\frac{F_{i}^{d}(t)}{M_{ii}(t)}$$

where *M_ii_*(*t*) is the inertial mass of agent *i*. The next search step involves identifying the values of the subsequent velocity and position of the agent. Therefore, its position and velocity can be calculated as follows:

(15) $$v_{i}^{d}(t+1)=rand_{i}\times v_{i}^{d}(t)+a_{i}^{d}(t)$$

(16) $$x_{i}^{d}(t+1)=x_{i}^{d}(t)+v_{i}^{d}(t+1)$$

where *rand_i_* is a random number in the interval [0, 1], and $v_{i}^{d}$ is the velocity of agent *i* in the *d*th dimension. This random number is employed to equip the search with a randomized feature. Gravitational and inertial masses are calculated using the fitness evaluation. A heavier mass equates to a more efficient agent. This indicates that superior agents have higher attraction and move more slowly. The gravitational and inertial masses are assumed to be equal, as displayed in the following:

(17) $$M_{ai}=M_{pi}=M_{ii}=M_{i},i=1,2,\cdots ,Nm$$

The values of agents are calculated using the fitting function. We can calculate the gravitational and inertial masses with the following equations:

(18) $$m_{i}(t)=\frac{fit_{i}(t)-worst(t)}{best(t)-worst(t)}$$

(19) $$M_{i}(t)=\frac{m_{i}(t)}{\sum_{j=1}^{Nm}m_{j}(t)}$$

where $fit_{i}(t)$ is the fitness value of agent *i* at time *t*. For a minimization problem, worst(t) and best(t) are defined as Equations (20) and (21), respectively: The summarized symbols of the GSA are listed in Table 2 for a simplified reading of the formulas

(20) $$best(t)=\underset{j\in \left\{1,\cdots ,Nm\right\}}{\min}fit_{j}(t)$$

(21) $$worst(t)=\underset{j\in \left\{1,\cdots ,Nm\right\}}{\max}fit_{j}(t)$$

The summarized symbols of the GSA are listed in Table 2 for a simplified reading of the formulas.

**Table 2:** Symbol summary for GSA.

Symbol	Explanation
$X_{i}=\left(x_{i}^{1},\cdots ,x_{i}^{d},\cdots ,x_{i}^{Nd}\right)$	position of agent *i*
$x_{i}^{d}$	*d*th dimension of $X_{i}$
Nd	dimension of an agent
Nm	number of agents
t	index of iteration
$t_{\max}$	total number of iterations
$V_{i}=\left(v_{i}^{1},\cdots ,v_{i}^{d},\cdots ,v_{i}^{Nd}\right)$	velocity of agent *i*
$v_{i}^{d}$	*d*th dimension of $V_{i}$
$F_{ij}^{d}(t)$	*d*th dimension of gravitational force where agent *j* acts on agent *i*
$F_{i}^{d}(t)$	total force that acted on agent *i* in *d*th dimension
G(t)	gravitational coefficient at time *t*
$G(t_{0})$	initial value of G(t)
α	a positive constant for G(t)
ε	a small positive constant for Equation (10)
$M_{aj}$	active gravitational mass related to agent *j*
$M_{pi}$	passive gravitational mass related to agent *i*
$R_{ij}(t)$	Euclidian distance between two agents *i* and *j*
*Kbest*	the set of first agents with larger mass
rand	random number in the interval [0,1]
$a_{i}^{d}(t)$	acceleration of agent *i* at time *t* and in *d*th dimension
$M_{ii}(t)$	inertial mass of agent *i*
$M_{i}(t)$	equality mass assumption for the gravitational and inertia mass for Equation (17)
$m_{i}(t)$	calculated variable for $M_{i}(t)$
$fit_{i}(t)$	fitting function (or fitness)
best(t)	strongest agent in the population
worst(t)	weakest agent in the population

**Figure 3:**
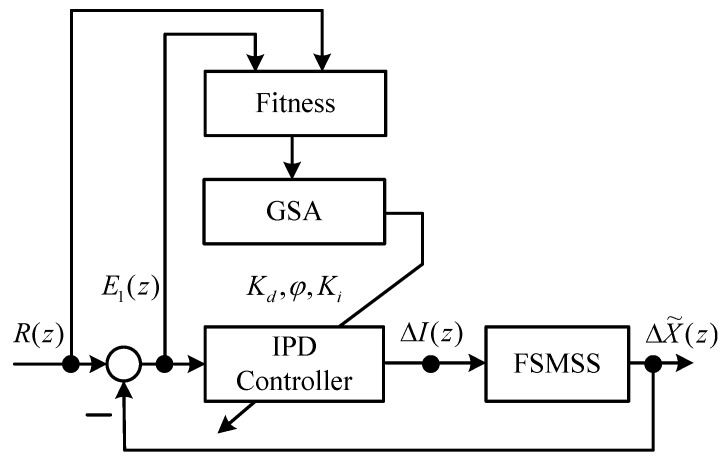
Block diagram of IPD–GSA control of a FSMSS.

**Table 3:** IPD–GSA searching procedure.

Procedure	Operation Details
Step 1:	Randomized initial controller parameters (*K_d_*, *ϕ*, *K_i_*) in the stable range Equations (6)–(7) of all agents. Set following parameters: Nd, Nm, $t_{\max}$, $G(t_{0})$, α, ε, and Kbest.
Step 2:	Execute the control system simulation (or experiment) for all agents of *t*-iteration.
Step 3:	Calculate fitness $fit_{i}(t)$.
Step 4:	Calculate formulae sequentially for Equations (20), (21), (18), (19), (17), (11), (12), (10), (13), (14), (15), and (16).
Step 5:	Update controller parameter position $X_{i}(t+1)$. Specify the stable range for Equations (6)–(7) of three controller parameters (*K_d_*, *ϕ*, *K_i_*).
Step 6:	Check the stopping criteria. If they are satisfied, then stop. Otherwise, proceed to Step 2.

**Figure 4:**
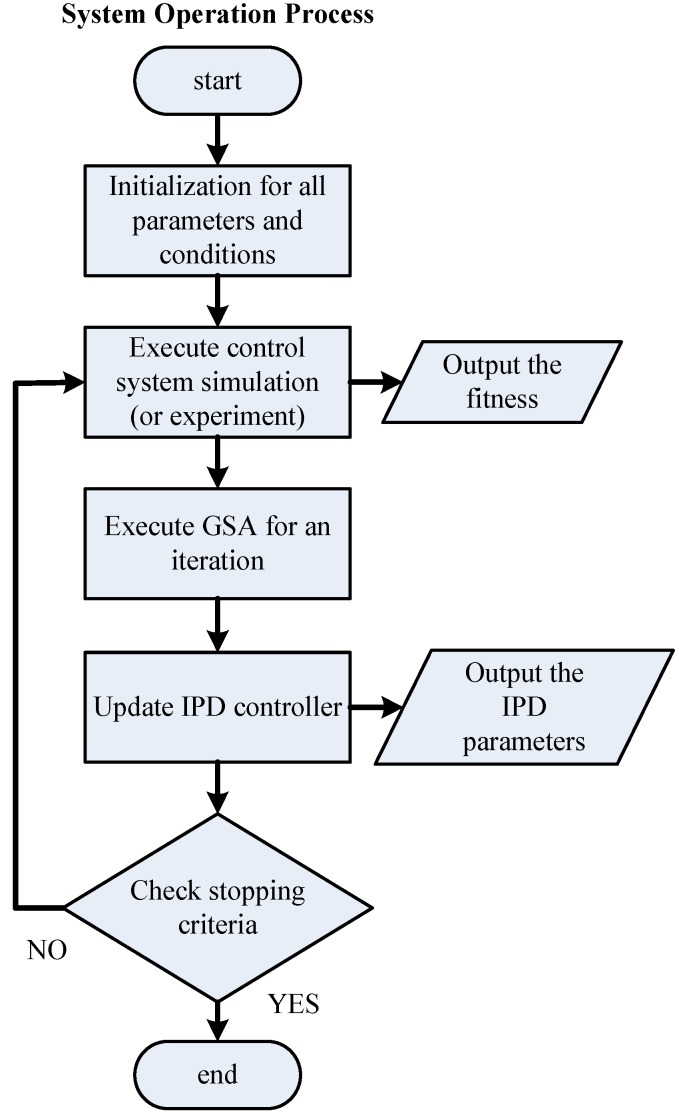
Flowchart of system operation process.

**Figure 5:**
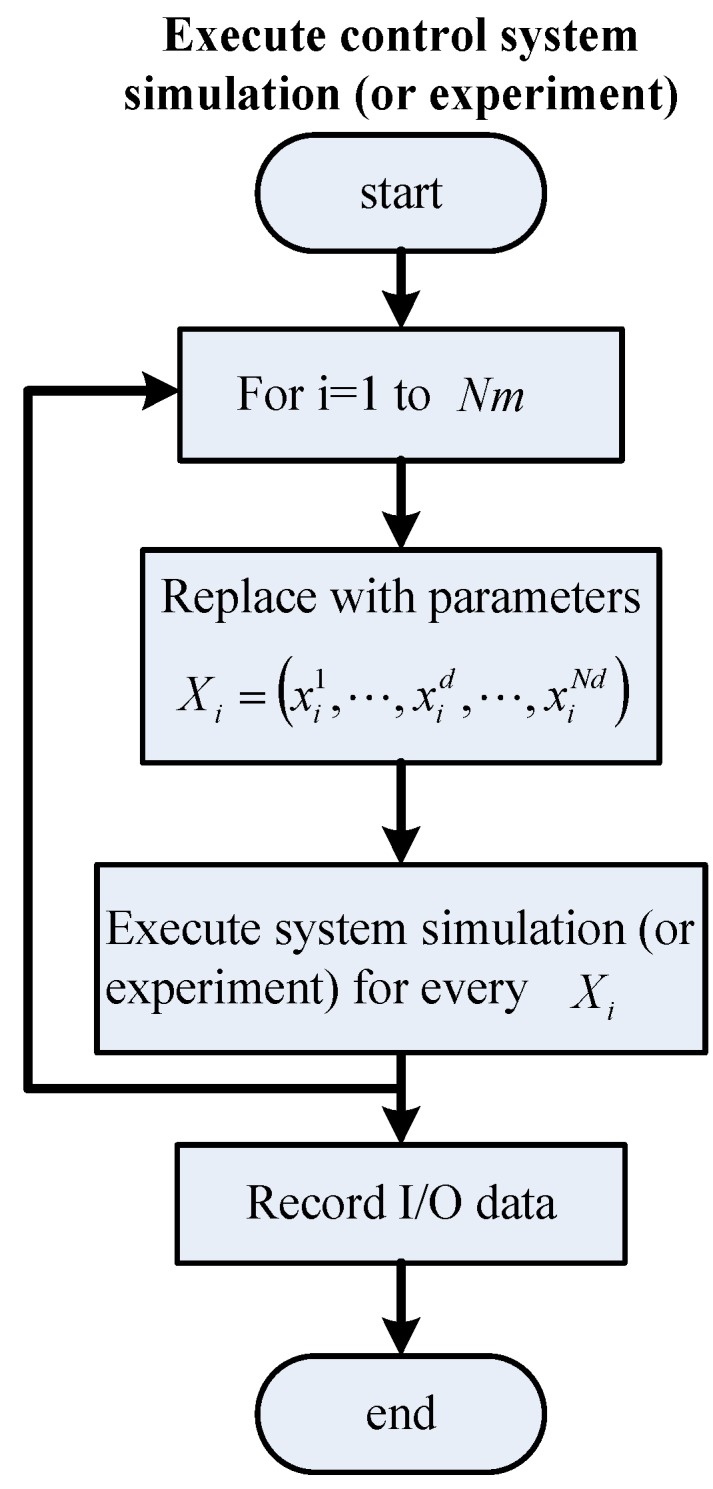
Flowchart for executing the control system simulation (or experiment).

**Figure 6:**
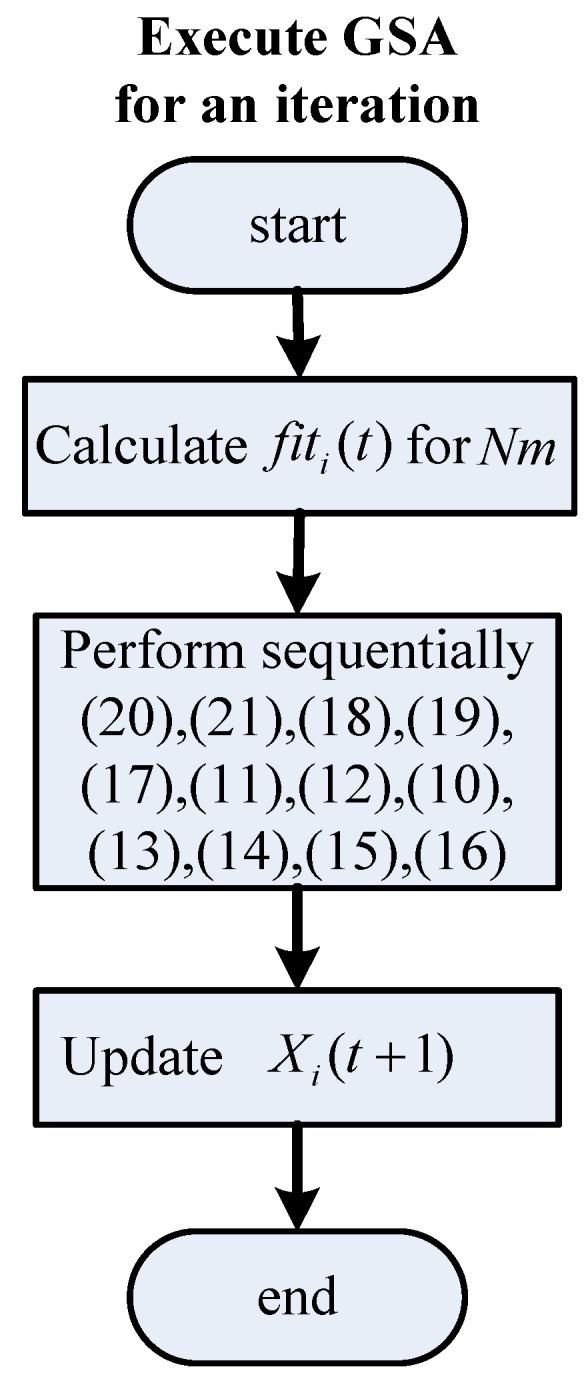
Flowchart for executing the GSA for an iteration.

The strategy of the GSA in tuning the IPD controller is provided in Figure 3. The parameter tuning of an IPD controller using the GSA can be accomplished by assigning the three parameters *K_d_*, *ϕ* and *K_i_* to enable the output response $\Delta \tilde{X}(z)$ to track reference input R(z). The search procedure of the proposed IPD–GSA control (IPD control by GSA tuning) is listed in Table 3. For explaining the proposed IPD–GSA control, three flowcharts were plotted, as displayed in Figure 4, Figure 5 and Figure 6. Figure 4 displays the system operation process. This process includes three main subprocesses: initialization, a control system simulation, and the GSA. A flowchart of the control system simulation is provided in detail in Figure 5. The program of the control system simulation is executed *N_m_* times for every iteration. The GSA flowchart is displayed in detail in Figure 6. First, the fitting functions are calculated for *N_m_* agents. Thereafter, the formulas of the GSA are calculated sequentially. Finally, the positions of agents are updated. The stable range of Equations (6)–(7) of the three controller parameters (*K_d_*, *ϕ*, and *K_i_*) is specified for every updated position.

## 4. Simulation

This section details the simulation of the proposed IPD–GSA control of the MSS. The difference equation of Equation (4) can be obtained as follows:

(22) $$\Delta \tilde{x}(k)=\tilde{\beta }\Delta \tilde{x}(k-1)-\Delta \tilde{x}(k-2)+\tilde{\sigma }\Delta i(k-1)$$

According to the material displayed in Figure 2, the difference equations of the variables $E_{1}(z)$, $E_{2}(z)$, $E_{3}(z)$, and ΔI(z) are expressed as follows:

(23) $$e_{1}(k)=r(k)-\Delta \tilde{x}(k)$$

(24) $$e_{2}(k)=e_{2}(k-1)+K_{i}e_{1}(k)$$

(25) $$e_{3}(k)=e_{2}(k)-\Delta \tilde{x}(k)$$

(26) $$\Delta i(k)=K_{d}e_{3}(k)+K_{d}\phi e_{3}(k-1)$$

where Equation (24) is an integrator, and Equation (26) is the PD controller. After solving Equations (22)–(26), we can simulate the IPD control of the MSS by using MATLAB software. The initial state conditions can be assumed to be zero.

The system parameters of the MSS are as follows: *β* = 2.002 and $\tilde{\sigma }=0.072$ [6]. The servo system requires an integrator for eliminating the steady-state error to step inputs. The IPD control (Figure 2) is this type of system. Because the state of the system is completely controllable, the desired closed-loop poles can be specified using the PD control loop. Next, the I control gain $K_{i}$ can be assigned to achieve optimal steady-state performance. The PD control is used to stabilize the MSS, as demonstrated in Figure 2. If the initial zero of the PD control is z=−ϕ=0.85, then the stable gain $K_{d}$ with respect to Equations (6) and (7) is approximated to:

(27) 0.1852<Kd<30.045 for ϕ=−0.85

Assuming that the initial zero z=−ϕ of the PD control is designed for $\tilde{\beta }=2.002$ and $\tilde{\sigma }=0.072$, the stable range of *K_d_* is obtained as follows:

(28) 0.0278(1+ϕ)<Kd<55.58(1−ϕ), and 0<Kd<−27.77ϕ

Conservatively, ϕ and $K_{i}$ are assigned as follows:

(29) −0.98≤ϕ≤−0.1, and 0.1≤Ki≤3.0

Therefore, Equations (28) and (29) are the design constraints of this simulation. 

The simulation of the IPD–GSA control is as follows. Let population size *N_m_* = 4. Additionally, let the dimension of each agent Nd = 3, with the position of *i*th agent as $X_{i}=\left(x_{i}^{1},x_{i}^{2},x_{i}^{3}\right)$. Each agent contains three controller parameters (*i.e*., *K_d_*, *ϕ*, and *K_i_*); in other words, $K_{d}=x_{i}^{1}$, $\phi =x_{i}^{2}$, and $K_{i}=x_{i}^{3}$. The design constraints of each agent are defined in Equations (28)–(29). The total number of iterations $t_{\max}$ is 100. The initial value of the gravitational coefficient $G(t_{0})$ is set as 100, and α is set to 20. A small positive constant ε is set to 10. The set of first agents with greater mass Kbest is set to the total number of agents (Nm). The fitness $fit_{i}(t)$ for the *i*’s iteration is assigned as defined in the following function:

(30) $$fit_{i}(t)=\gamma_{1}\cdot Mp+\gamma_{2}\cdot \sum_{k=1}^{100}e_{1}^{2}(k)$$

where *M_p_* is the peak overshoot, $\gamma_{1}=10$ is the weighting factor of *M_p_*, and $\gamma_{2}=10$ is the weighting factor of the integral squared error. 

**Figure 7:**
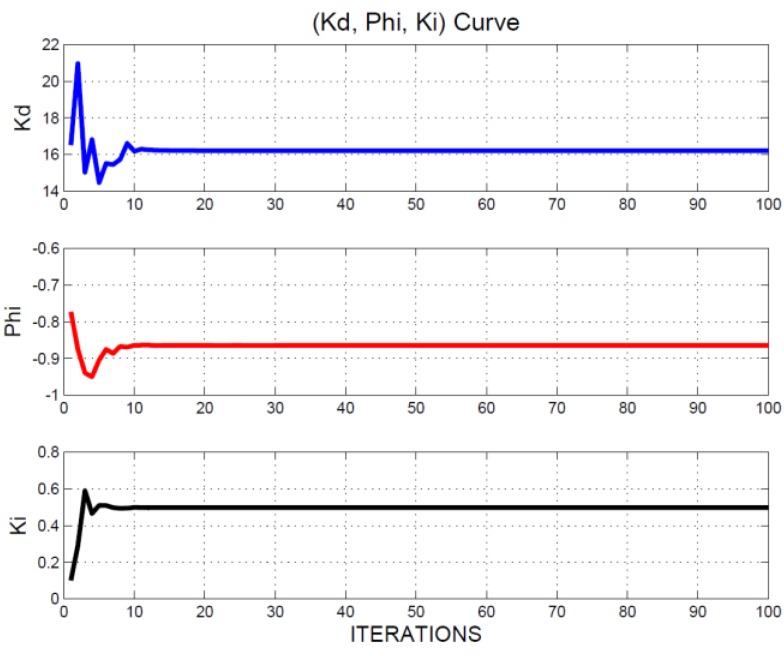
(*K_d_*, *ϕ*, *K_i_*) iterative curve.

**Figure 8:**
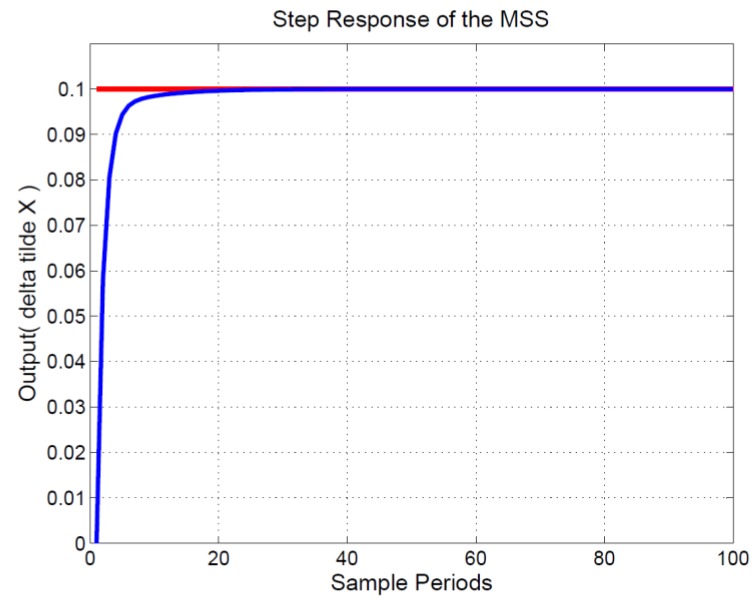
Step response of the MSS.

Both *M_p_* and $\sum_{k=1}^{100}e_{1}^{2}\left(k\right)$ of Equation (30) are calculated from system responses of Equations (22)–(26) for the *i*’s iteration. The IPD controller (*K_d_*, *ϕ*, *K_i_*) of *i*’s iteration is $X_{i}=\left(x_{i}^{1},x_{i}^{2},x_{i}^{3}\right)$. The simulations were performed using MATLAB software. The (*K_d_*, *ϕ*, *K_i_*) iterative curve is provided in Figure 7. After 100 iterations, the position of four agents approximated X=[16.2138,−0.8646,0.4972]. Optimal fitness (*best*(*t*)) approximated 0.1230. Therefore, the optimal IPD controller could be obtained as follows: $K_{d}=16.2138$, ϕ=−0.8646, and $K_{i}=0.4972$. The output response is displayed in Figure 8; the horizontal axis represents the sampling time, the unit is 1 mini-s, and the vertical axis is the measured output ($\Delta \tilde{x}$) of the MSS. The red line represents the step input (r), the amplitude of which is 0.1 units. The blue curve indicates the measured output ($\Delta \tilde{x}$) of the MSS. As displayed in Figure 8, the steady-state error and overshoot to a step input were absent. For the MSS, this indicated strong performance.

## 5. Experiments and Results

**Figure 9:**
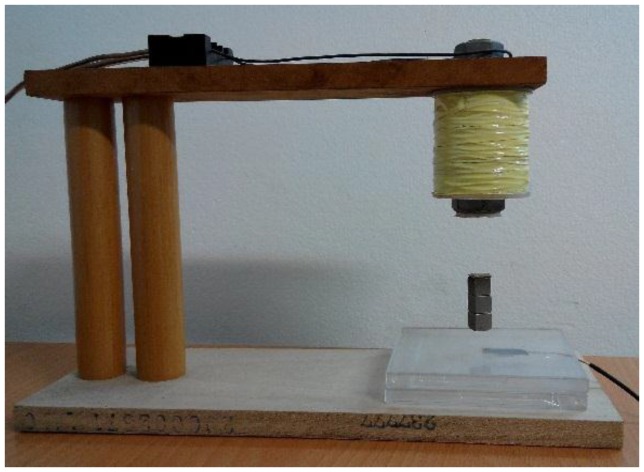
Picture of a FSMSS.

**Figure 10:**
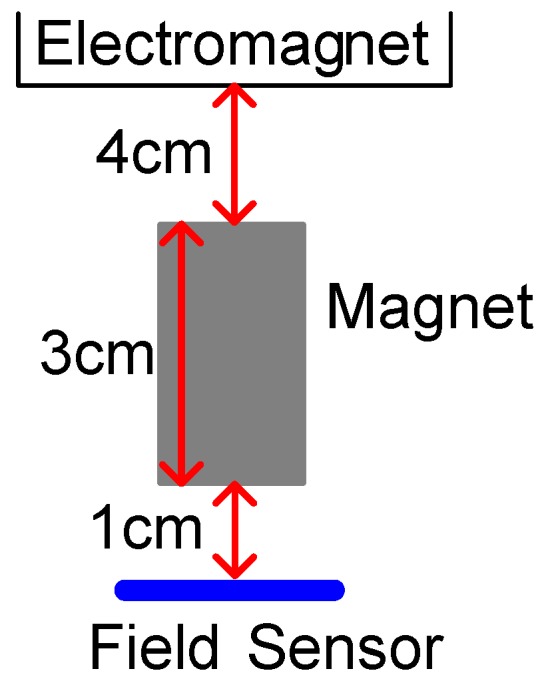
Sketch of magnet position.

This section details the hands-on experiments. The FSMSS apparatus is provided in Figure 9 [6]. A sketch of the magnets, a field sensor, and an electromagnet is displayed in Figure 10. The suspended object (magnet) was composed of three 1 cm cubes as shown in Figure 9, and weight of a cube is 7 grams. The material of the magnet is NdFeB. The magnetic core of the electromagnet is a bolt with nut, and the material is steel. The shape of the magnetic core is cylindrical. The inner diameter of the magnetic core is 15 mm and outer diameter is 50 mm. The height is 55 mm. The Hall Effect device can sense the strength of the magnetic field of the three-cubed magnet. Therefore, this device serves as the position sensor. This MSS apparatus is the FSMSS. Hall Effect devices are superior in position sensing to optical [2,7] and electromechanical sensing devices in the MSS. If the position measurement is acquired through optical position sensors, then it is disturbed easily by surrounding light sources. The magnet position sensor is installed on the bottom of the frame. Because the magnet is placed relatively near the position sensor, which is situated far from the solenoid, the electromagnet does not affect the output measurement. The main focus of this study was the application of a magnetic sensor. Thus, a magnetic field sensor was utilized to measure the position of the suspended object. In this apparatus, the position sensor is SS495A [6].

**Figure 11:**
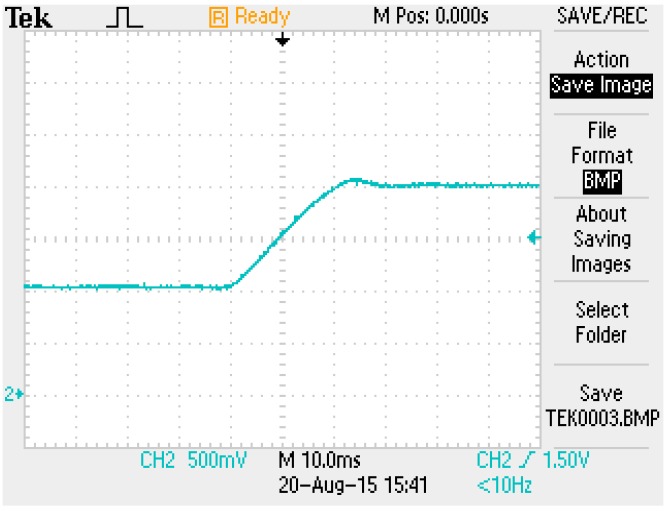
Step response of the power amplifier (current driver).

A power amplifier provides a current that passes through the electromagnetic coil. When a current passes through the electromagnet, a magnetic force is generated. The strength of the generated magnetic force is proportional to the square value of the current through the coil [2,6,7]. The FSMSS uses magnetic attraction to pull a magnet upward, against gravity. To control the current of the electromagnet, a current transducer is employed to measure the coil current. A current transducer is a Hall Effect current sensor with internal integrated circuits. In this apparatus, the current transducer is LA55-P [6]. The performance of the power amplifier (serving as a current driver) is provided in Figure 11. The step response of the coil current was from one to two units. A steady-state error was absent in the power amplifier. The settling time was approximately 25 ms. Therefore, the electromagnetic coil current was controlled effectively. 

**Figure 12:**
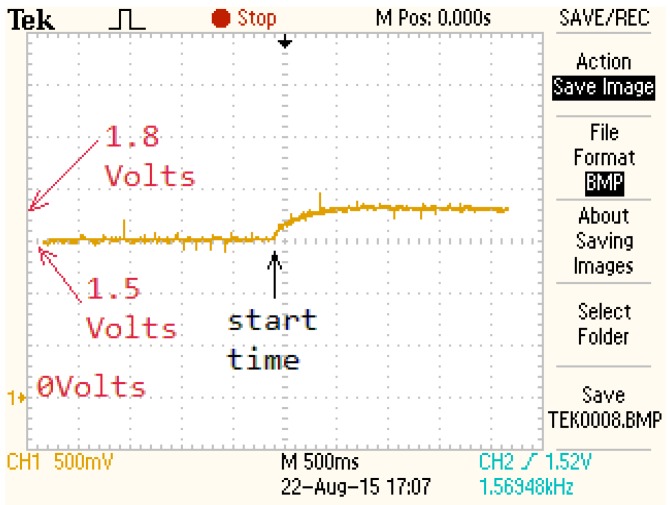
Output response of a FSMSS.

The measured output response of the FSMSS is provided in Figure 12. The voltage of equilibrium was 1.5 V. The input voltage was from 1.5 to 1.8 V. The optimally tuned IPD controller in the previous section was employed in this hands-on experiment. The orange curve in Figure 12 depicts the output measurement and represents the output of the SS495A circuit, which ranged from 1.5 to 1.8 V. The steady-state error and system overshoot did not occur in this experiment. The setting time was approximately 0.5 s. For the FSMSS, this result indicates strong performance.

## 6. Conclusions

The main contribution of this study is the GSA-tuning IPD control for MSSs. This IPD control can stabilize the MSS with the provided constraints. If the conditions are stabilized, then the integrator can eliminate the steady-state error to a step input. An IPD controller has three parameters and can be tuned to achieve optimal performance under the stable condition. The tuning method in this study was the GSA. The advantages of IPD–GSA are as follows: the GSA is based on heuristics, simplicity, and an absence of assumptions. This paper details all of the simulations and hand-on experiments that were conducted. According to the results, the proposed control scheme is appropriate for the FSMSS. 
